# Transforming Pediatric Urology With Artificial Intelligence: A Narrative Review of Current Evidence and Practice

**DOI:** 10.7759/cureus.100893

**Published:** 2026-01-06

**Authors:** Muhammad Rakib Hasan, Angelos Christofides, Pradip Subedi, Momen Abdelglil, Alaa Alghanemi, Kawthar Shehab

**Affiliations:** 1 Urology, Watford General Hospital, West Hertfordshire Teaching Hospitals NHS Trust, Watford, GBR; 2 Urology, West Hertfordshire Teaching Hospitals NHS Trust, Watford, GBR; 3 Urology, Northwick Park Hospital, London North West University Healthcare NHS Trust, Harrow, GBR; 4 Pediatric Surgery, Mansoura University Children Hospital, Mansoura, EGY; 5 Internal Medicine, Faculty of Medicine, Mansoura University, Mansoura, EGY

**Keywords:** artificial intelligence, clinical decision support systems, deep learning, hydronephrosis, hypospadias, machine learning, neural networks, pediatric urology, posterior urethral valves, vesicoureteral reflux

## Abstract

The use of artificial intelligence (AI) with pediatric urology is reshaping clinical care by enhancing diagnostic accuracy, surgical planning, and postoperative monitoring. AI integration offers significant advancements in managing conditions like hydronephrosis, where deep learning models automate severity grading and predict obstruction more accurately than conventional methods. In penile anomalies, such as hypospadias, AI reduces subjectivity through standardized image analysis, aiding in diagnosis and surgical decision-making. Furthermore, machine learning algorithms assist in risk-stratifying patients with vesicoureteral reflux to optimize antibiotic prophylaxis and predict resolution. Beyond diagnostics, AI enhances surgical precision in procedures like pyeloplasty through 3D simulation and real-time intraoperative navigation. However, widespread adoption is currently limited by challenges such as data scarcity, strict privacy regulations, and the need for ethical legal frameworks regarding patient data. Future developments must focus on creating transparent, interpretable decision support systems that can safely integrate into standard clinical workflows.

## Introduction and background

Rapid technological advancements in medicine, combined with digital patient record systems and increased computational capabilities, are driving the generation of large-scale medical datasets [1]. As the quantity, diversity, and accessibility of big data in medical practice expand, clinicians encounter increased complexity in integrating this information to formulate accurate diagnoses, customize treatment approaches, and forecast patient outcomes [2].

The integration of artificial intelligence (AI) has emerged as a focal point in contemporary medicine. This trend is particularly evident in the field of urology, which has witnessed an exponential surge in research publications utilizing AI methodologies in recent years [3,4]. AI has become widely integrated into urologic practice, fundamentally shifting how clinical data is utilized. When compared to conventional statistical methods, AI approaches demonstrate superior predictive accuracy and offer a more robust capacity for the explorative analysis of large, complex data cohorts [4].

Machine learning (ML) has evolved for decades, but recently advanced rapidly thanks to better hardware, software, and larger datasets. In healthcare, ML can analyze electronic records, medical images, and biomedical data to detect complex hidden patterns. This helps identify risk factors, guide treatment decisions, improve monitoring, and predict prognosis. Unlike many traditional methods, ML often avoids strict statistical assumptions and improves through continual learning [3]. Advanced clinical models utilizing ML technology are being integrated into urological practice to facilitate the identification of urological pathologies, identify anomalous imaging findings, and anticipate patients' future clinical outcomes [5].

Urologists have implemented ML models to predict cancer-related outcomes, select the most suitable patients for functional urological surgery, and forecast stone elimination in endourological treatments [6-8]. In this narrative review, we aim to summarize the present evidence regarding the application of AI in pediatric urology, specifically focusing on its utility in diagnosis and outcome prediction. We also seek to identify the barriers to implementation, including data scarcity, and propose future directions for integrating these technologies into standard clinical practice.

## Review

Methodology

We conducted a narrative review article in which a literature search was conducted to identify relevant studies investigating the application of AI and ML within the field of pediatric urology. Electronic databases, including PubMed/MEDLINE, Embase, and Google Scholar, were queried from inception through December 2025. The search strategy utilized combinations of Medical Subject Headings (MeSH) terms and free-text keywords, including "artificial intelligence," "machine learning," "deep learning," "neural networks," "pediatric urology," "hydronephrosis," "vesicoureteral reflux," "posterior urethral valves," and "clinical decision support systems." Boolean operators (AND, OR) were employed to refine search results and ensure the retrieval of clinically relevant literature.

The review prioritized original research articles, prospective and retrospective cohort studies, and systematic reviews published in the English language. Inclusion was limited to studies that specifically developed, validated, or applied computational algorithms for the diagnosis, prognosis, or management of pediatric urologic conditions. Studies were excluded if they focused exclusively on adult populations, lacked specific descriptions of the ML methodologies used, or were available only as abstracts without sufficient data for evaluation.

Titles and abstracts were initially screened for relevance, followed by a full-text review of selected articles. Reference lists of included studies were manually cross-referenced to identify additional pertinent literature. Data were extracted and synthesized thematically, categorizing findings by specific urologic pathology - specifically, hydronephrosis, vesicoureteral reflux (VUR), and posterior urethral valves (PUV) - to provide a structured narrative overview of the current evidence, methodological approaches, and clinical implications of AI integration in this domain.

Value of AI for pediatric urology

Pediatric urology manages a wide range of genitourinary conditions in children, from routine infections to complex congenital defects and cancers, requiring a coordinated, multidisciplinary team of specialists to ensure optimal patient care [9]. In recent years, the integration of AI into pediatric urology has demonstrated significant potential to enhance the entire continuum of care, offering improvements in diagnostic accuracy, preoperative planning, surgical execution, and postoperative monitoring [10].

By synthesizing high-dimensional data from medical imaging and patient records, AI systems contribute significantly to the early detection and improved diagnostic accuracy of urological disorders in children [11]. AI algorithms also enable the formulation of tailored therapeutic strategies by systematically analyzing individual patient characteristics, thereby optimizing clinical efficacy and minimizing potential complications [12]. AI-integrated monitoring platforms facilitate the continuous surveillance of pediatric patients, capable of generating real-time notifications to alert clinicians to any deviations from the expected clinical trajectory [12].

In the intraoperative setting, AI-augmented surgical systems enhance procedural precision and safety. By refining surgical execution, these technologies serve to minimize perioperative complications and accelerate postoperative convalescence in the pediatric population [3].

**Figure 1:**
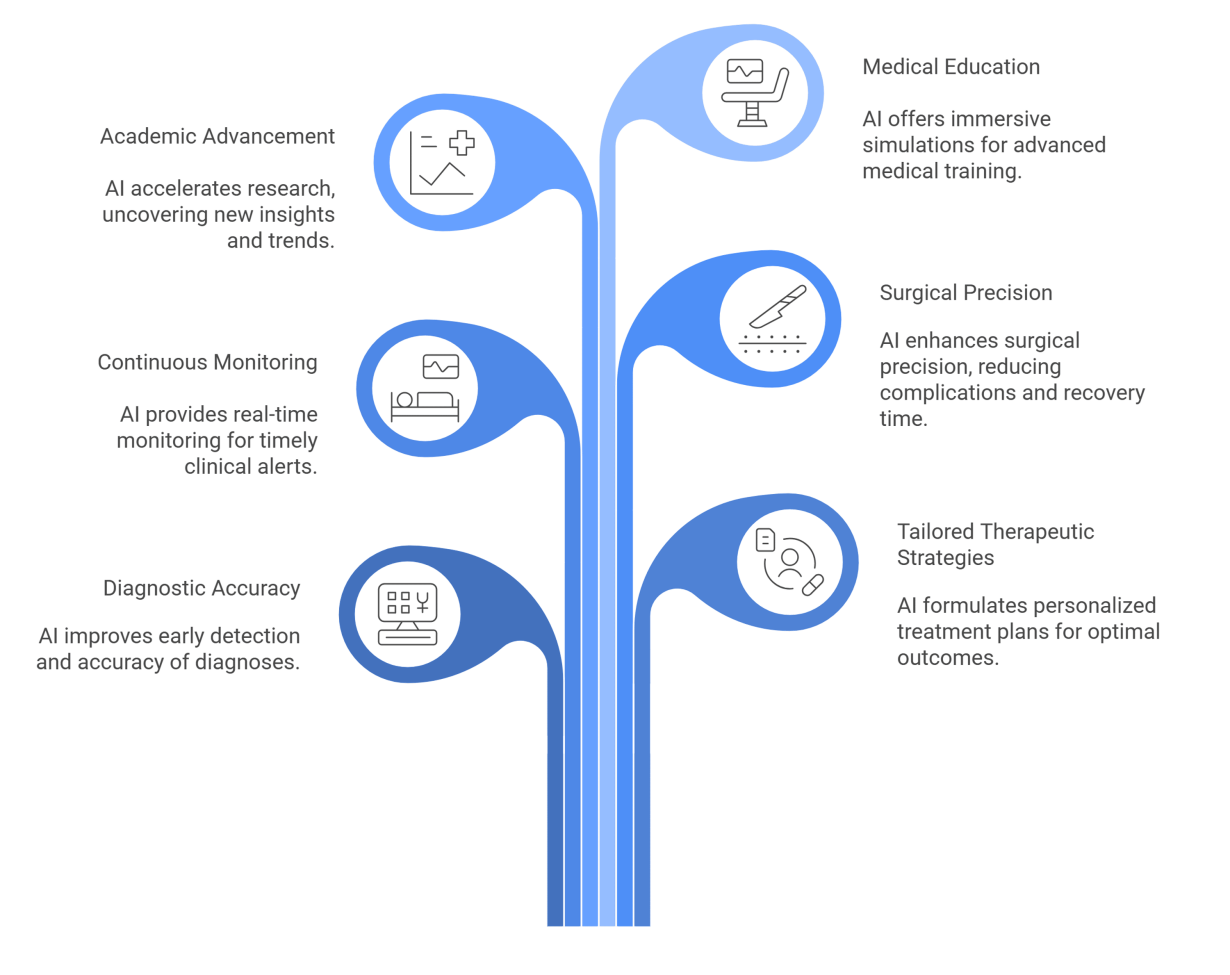
Unveiling AI's impact on pediatric urology. Figure Credit: The authors. Source: References [10-14].

Beyond direct clinical application, AI accelerates academic advancement by interrogating large-scale datasets to uncover latent trends and novel pathophysiological insights. Furthermore, in the domain of medical education, AI-driven simulations and virtual reality environments provide immersive, high-fidelity resources essential for the training of residents and pediatric urologists [13]. AI is fundamentally reshaping surgical education by enabling pediatric urology trainees to acquire and refine skills outside the traditional operating room. Through high-fidelity virtual reality simulations, these systems provide immersive environments where residents can rehearse complex maneuvers and simulate intricate clinical scenarios. This risk-free, controlled setting is essential for building the technical proficiency and confidence required to successfully perform pyeloplasty (Figure 1) [14].

Hydronephrosis

Hydronephrosis is characterized by the accumulation of urine, resulting in the dilation of the renal collecting system. It represents the most prevalent neonatal urinary tract anomaly, affecting approximately 1% to 5% of all newborns [15]. Typically identified via prenatal sonography, hydronephrosis may stem from a spectrum of underlying etiologies, most notably ureteropelvic junction obstruction (UPJO) and VUR [16]. While a significant proportion of cases undergo spontaneous resolution, severe manifestations necessitate surgical correction. In these high-risk scenarios, timely intervention is critical, as failure to treat can lead to irreversible deterioration of renal function [16-19].

The subjective interpretation of renal sonography often limits the conventional assessment of hydronephrosis severity. Deep learning, characterized by its data-driven algorithmic capacity to classify complex imagery, has emerged as a promising methodology to standardize and objective this grading process [20].

ML has been effectively applied to renal ultrasound imaging to stratify hydronephrosis severity, detect obstruction, and prognosticate long-term renogram outcomes. Notably, Smail et al. utilized a deep learning model that processes sagittal ultrasound views to automatically classify hydronephrosis according to the Society of Fetal Urology (SFU) grading system (grades I-IV) [20]. The model demonstrated performance comparable to physicians, particularly in distinguishing SFU grades II and III - categories known for significant inter-observer variability - achieving an overall accuracy of 71%. Similarly, Erdman et al. utilized both sagittal and transverse ultrasound views to differentiate between obstructive and physiologic hydronephrosis; their model yielded an accuracy of 58% but demonstrated high discriminative ability with an area under the curve (AUC) of 0.93 [21].

Cerrolaza et al. also employed renal ultrasound imaging, but in this study, the researchers manually preprocessed the images and segmented the kidneys. From these images, they extracted 131 variables describing kidney size, geometric form, and curvature. These parameters were then applied to predict T1/2 thresholds, yielding better results than the SFU grading system. The model showed potential to safely reduce the need for diuretic renograms in 62% of pediatric cases [22].

In a related study, Blum et al. [23] employed a Support Vector Machine (SVM) algorithm to predict obstruction, utilizing 43 numeric variables extracted from drainage curves. The model pinpointed tracer clearance at 30 minutes after furosemide administration as the primary predictive factor. Unlike image-based approaches requiring pre-processing, this method allows for the direct input of recorded values from standardized renograms. Nevertheless, future research must focus on external validation to confirm the clinical utility of these predictive tools.

Erdman et al. [13] transformed treatment paradigms by introducing deep learning algorithms capable of predicting obstructive hydronephrosis based exclusively on ultrasound imaging. The high accuracy of these models facilitates the precise identification of high-risk patients requiring surgical intervention, thereby reducing the burden of unnecessary invasive diagnostics and prolonged follow-up. Moreover, the deployment of such tools promotes standardized management and consistent diagnosis, a benefit particularly vital for remote regions lacking specialized pediatric urologic care.

Lorenzo et al. [24] reported 87% accuracy in predicting surgical intervention for prenatal hydronephrosis using clinical variables alone. Although the study highlights the potential of using readily available clinical databases, it was limited by a lack of transparency in model development and the use of subjective surgical indications as the outcome measure.

Pyeloplasty

Pyeloplasty remains the gold standard for treating UPJO in children. Although clinical outcomes are historically excellent, the procedure presents unique technical challenges due to the diminutive size and delicate nature of pediatric anatomy [10].

AI enhances preoperative strategies in pediatric pyeloplasty by converting standard imaging into detailed 3D models for simulation. These tools allow surgeons to determine the optimal approach based on the unique anatomical variations of each child, ensuring a highly personalized surgical plan [25].

Intraoperative surgical navigation systems enhance operative precision by integrating AI with real-time imaging data. By superimposing critical information, such as vascular maps, anatomical landmarks, and planned trajectories, directly onto the surgical field, these systems facilitate complex tasks like dissection and suturing. Consequently, this augmented visualization minimizes intraoperative errors and optimizes overall surgical outcomes [10]. AI systems have the capacity to analyze complex intraoperative variables, including instrument kinematics, tissue characteristics, and physiological parameters, to detect subtle deviations from optimal surgical dynamics. By alerting surgeons to potential hazards or departures from standard techniques, these systems provide proactive decision support. This enhanced situational awareness facilitates timely interventions, thereby mitigating operative risks and optimizing patient outcomes [26].

The future of pediatric pyeloplasty lies in the utilization of AI to tailor interventions to individual anatomical requirements and provide dynamic intraoperative support. The implementation of advanced algorithms and imaging modalities within surgical platforms is expected to drive higher accuracy and lower complication rates. Concurrently, AI-powered virtual reality curricula will revolutionize surgical education, ensuring that the next generation of urologists is better equipped to deliver high-quality patient care [27].

Vesicoureteral reflux (VUR)

Keskinoglu et al. trained a neural network to identify VUR in children with uncomplicated urinary tract infections (UTIs), integrating data from physical exams, labs, and renal ultrasound. Despite the multimodal input, the model demonstrated low specificity. The authors suggest that the inconsistent manifestation of VUR within these standard clinical variables makes accurate prediction difficult without direct voiding cystourethrography [28]. Similarly, Logvinenko et al. utilized a neural network to predict VUR severity. Their findings emphasized that features typically significant in traditional regression (e.g., circumcision status, UTI history) do not necessarily translate into effective inputs for predictive ML models [29].

Two distinct modeling approaches have been applied to determine VUR grades from voiding cystourethrograms (VCUG). Eroglu et al. utilized a hybrid deep learning model based on raw VCUG images, achieving a perfect AUC of 1.0. However, the clinical applicability of these findings is debated due to the inclusion of normal (non-refluxing) VCUGs in the dataset and a lack of rigorous validation. In contrast, Khondker et al. employed a feature-based approach, extracting four specific annotations - ureteral tortuosity and diameter at the proximal, distal, and maximal points. This model achieved a lower AUC of 0.83 for predicting high-grade (≥grade IV) VUR [30,31].

Kirsch et al. [32] leveraged random forest algorithms to select variables for "VURx," a hybrid clinical scoring tool designed to predict primary VUR resolution. Similarly, Seckiner et al. [33] utilized demographics and treatment type to forecast outcomes. In the context of endoscopic intervention, Serrano-Durbá et al. [34] validated the superiority of ML over simple regression; their study showed that ML effectively modeled the complex interactions between variables (e.g., age, sex, etiology) that traditional regression analysis failed to capture.

Pediatric penile conditions

Hypospadias

Hypospadias represents the most common congenital anomaly affecting the male external genitalia globally [35-37]. Despite the condition's prevalence, the diagnosis of hypospadias frequently precipitates significant parental anxiety, often exacerbated by uncertainty regarding the nature of the pathology and the complexity of available medical information [38]. Furthermore, the availability of capable multidisciplinary teams for diagnosis and surgery is severely limited, especially in resource-constrained environments [39]. To standardize the assessment of severity, several validated scoring systems have been established, including the Glans-Meatus-Shaft (GMS) score, the Meatus-Chordee-Glans-Urethral Plate Quality (MCGU) system, and the Hypospadias Objective Penile Evaluation (HOPE) score [40,41].

Wahyudi et al. presented a protocol to develop a digital pattern recognition system and mobile application for diagnosing hypospadias. Addressing specialist shortages in Indonesia, they aim to train an artificial neural network (ANN) using parent-captured images to identify various critical anatomical parameters, such as meatal location and glans shape. This tool seeks to empower non-specialists and families in low-resource settings, effectively standardizing diagnosis and potentially improving management outcomes through the implementation of accessible, user-friendly AI technology [36]. However, previous research highlights significant barriers to the clinical adoption of mobile health applications, particularly regarding the need to demonstrate tangible utility to practitioners [42].

Fernandez et al. aimed to reduce subjectivity in hypospadias classification by utilizing ML for image recognition. Identifying that anatomical assessment often varies among clinicians, they trained a model using TensorFlow on a database of 1,169 standardized images of distal and proximal hypospadias. The model’s performance was validated against expert pediatric urologists using a set of 29 test images. Results showed the algorithm achieved 90% accuracy, closely mirroring the "almost perfect" inter-rater agreement (kappa = 0.86) observed among human experts. The authors concluded that their deep learning model successfully emulates expert classification. This study suggests that AI technologies hold significant promise for standardizing hypospadias assessment and could eventually incorporate complex variables to improve the prediction of surgical outcomes in pediatric urology [43].

Abbas et al. sought to enhance the objectivity of hypospadias assessment by automating the Plate Objective Scoring Tool (POST). Utilizing a dataset of 691 images, they developed a deep convolutional neural network (CNN) capable of localizing the glans and identifying five critical urethral plate landmarks. The model demonstrated exceptional performance, achieving a mean average precision of 99.5% in glans detection and a minimal error rate in landmark placement. This study confirms that deep learning can effectively standardize the quantification of urethral plate characteristics, offering a robust alternative to subjective manual scoring [44].

Penile Curvature (PC) and Circumcision

Wahyudi et al. developed an ANN to assess penile anatomy and determine eligibility for circumcision. By training the model on parent-captured images from dorsal, lateral, and ventral perspectives, the researchers achieved high classification accuracy, ranging from 88.89% to 92.5%. This cloud-based system employs transfer learning to mitigate limited dataset challenges and offers a scalable solution for large-scale screening, particularly valuable in resource-limited settings for identifying contraindications before surgery [45].

Abbas et al. validated an AI-based tool for automated PC measurement using 3D-printed models. Comparing the AI application against visual inspection and goniometry among 35 practitioners, the study demonstrated that the AI method achieved the lowest mean absolute error and highest inter-rater reliability. With a measurement time of 22 seconds, the authors concluded that the AI model offers a more practical, consistent, and accurate alternative to traditional assessment tools for guiding hypospadias management [46].

Limitations and future directions

The application of AI within the field of pediatric urology faces significant hurdles, primarily regarding the acquisition of high-quality data. Obtaining sufficiently large and diverse datasets is often impeded by strict privacy regulations, the inherently limited size of pediatric populations, and the rarity of specific urologic pathologies. These constraints frequently force researchers to rely on small sample sizes, which heightens the risk of overfitting. In such scenarios, ML models may hyper-specialize to the training data, resulting in poor generalization to new cases and unstable performance where apparent success may merely be a statistical artifact of chance rather than the identification of genuine clinical patterns. To counteract these methodological weaknesses, researchers have proposed strict validation standards, including the reporting of confidence interval estimates to better quantify uncertainty [3,47-49].

The integration of AI into pediatric urology necessitates a rigorous examination of the associated ethical and legal frameworks. Foremost among these challenges are patient privacy and data stewardship. Since AI algorithms require the assimilation of massive datasets - comprising sensitive genetic, imaging, and clinical records - they introduce significant risks regarding informed consent and data security. Consequently, a major hurdle in the era of AI-driven healthcare is striking a balance between safeguarding patient confidentiality and leveraging data to foster scientific innovation [3]. AI introduces ethical complexities that extend beyond patient care to include algorithmic liability and economic impact. For pediatric urologists, navigating these issues requires strong interdisciplinary partnerships and clear professional guidelines. Such measures are vital for fostering responsible innovation that prioritizes the welfare of both patients and society [5].

Future investigation is required to effectively integrate AI into multimodal management strategies for pediatric urological conditions. Specifically, research should prioritize the development of AI-driven decision support systems that synthesize patient-specific variables to optimize treatment planning and identify the most efficacious therapeutic regimens [23]. A critical imperative involves improving the interpretability and transparency of AI architectures within pediatric urology. Prioritizing these aspects is essential for cultivating clinical trust and fostering robust collaborative synergies between computational experts and healthcare providers [50].

**Figure 2:**
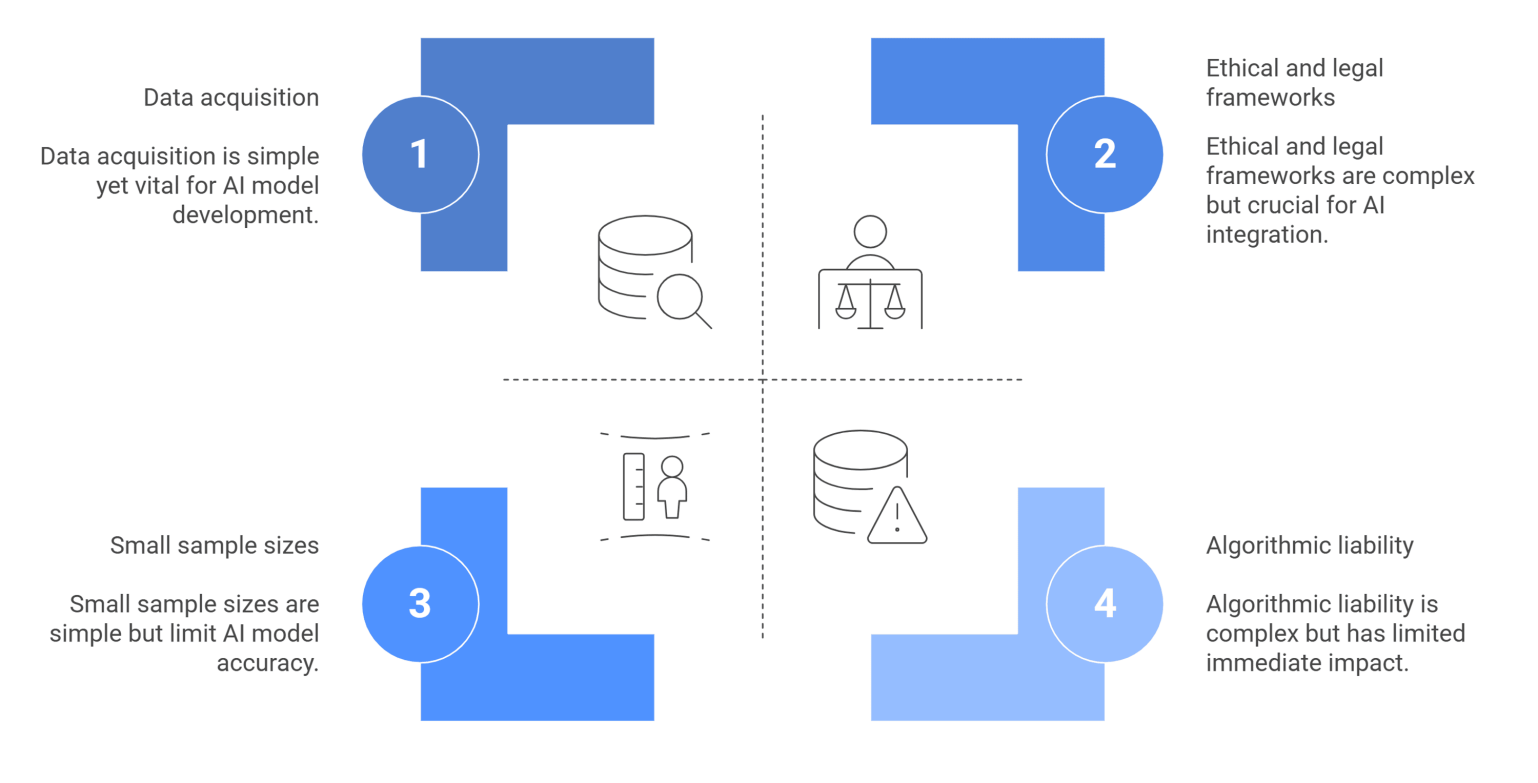
Challenges in the use of AI in pediatric urology. Figure Credit: The authors. Source: References [3,47-49].

The integration of AI has emerged as a dominant frontier in modern medicine. Unlike conventional statistical methods, which are often constrained by rigid assumptions, ML algorithms excel at deciphering intricate data patterns to generate robust predictive models. This technical advantage has catalyzed an exponential surge in pediatric urology literature utilizing AI methodologies. However, while these advancements hold immense promise for improving disease management, maintaining a trajectory of high-impact research requires a pragmatic assessment of the unique challenges inherent to pediatric urological practice (Figure 2) [3].

The field of AI in pediatric urology is rapidly evolving. Recent research has increasingly focused on various areas, including pediatric oncology, organ transplantation, and the management of kidney stones. This growth in studies highlights the potential for AI to enhance diagnosis and treatment in these critical areas of children's health. As technology advances, we can expect even more innovative applications of AI in improving outcomes for young patients facing these challenges [51,52].

ML holds great promise for improving the quality of care for patients in the field of pediatric urology. While the advancements in AI are indeed thrilling, the current scarcity of robust evidence creates challenges in implementing these models effectively in clinical practice. To move forward, it is crucial to establish thorough reporting standards and prioritize the development of high-quality models [53].

## Conclusions

The integration of AI into pediatric urology represents a paradigm shift, offering diagnostic and predictive capabilities that far exceed the limitations of traditional statistical methods. By leveraging ML to decipher complex data patterns, AI is actively transforming the continuum of care - from automating the grading of hydronephrosis and optimizing surgical planning in pyeloplasty to revolutionizing trainee education through virtual reality simulation. These technologies hold the potential to standardize clinical assessments, significantly reduce inter-observer variability, and facilitate highly personalized treatment strategies that improve overall patient outcomes.

Despite this promise, the path to widespread clinical adoption requires a pragmatic approach to overcome significant challenges, particularly regarding data scarcity, patient privacy, and the need for algorithmic transparency. Future success will depend on the development of robust, multimodal decision support systems and the establishment of ethical frameworks that prioritize patient safety and data security. Ultimately, fostering interdisciplinary collaboration between clinicians and AI developers is essential to ensure these innovations are not only technically sound but also clinically interpretable and impactful for the pediatric population.
